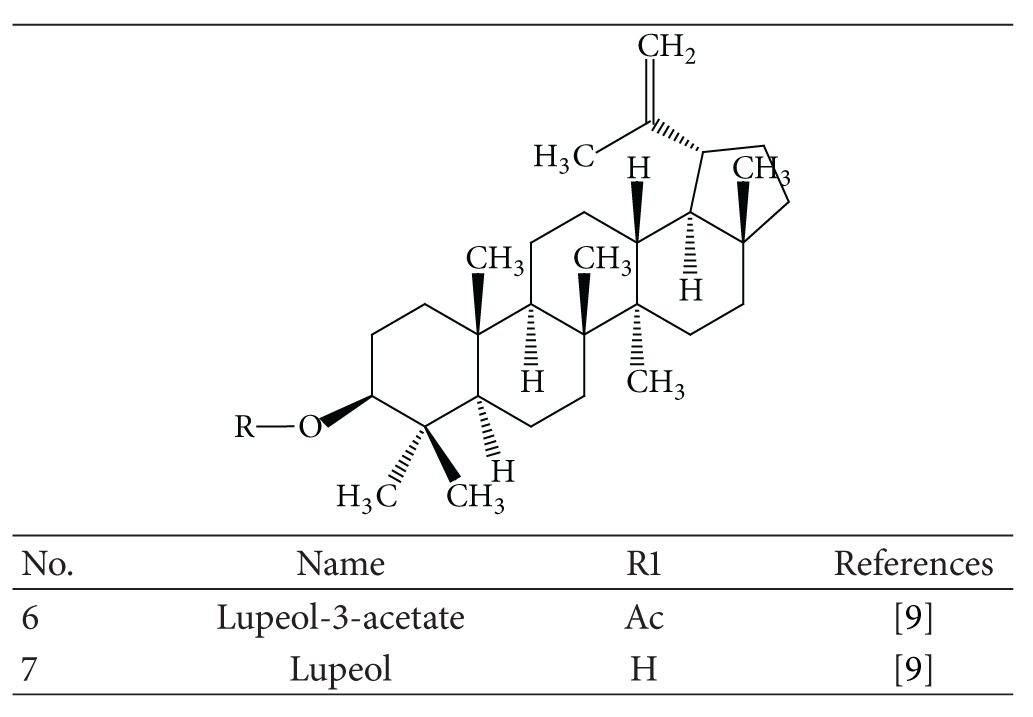# *Mallotus philippinensis* Muell. Arg (Euphorbiaceae): Ethnopharmacology and Phytochemistry Review

**DOI:** 10.1155/2014/213973

**Published:** 2014-07-08

**Authors:** Mayank Gangwar, R. K. Goel, Gopal Nath

**Affiliations:** ^1^Laboratory of Gastrointestinal Infections and Molecular Diagnosis, Department of Microbiology, Institute of Medical Sciences, Banaras Hindu University, Varanasi 221005, India; ^2^Department of Pharmacology, Institute of Medical Sciences, Banaras Hindu University, Varanasi 221005, India

## Abstract

*Mallotus philippinensis* Muell. Arg (Euphorbiaceae) are widely distributed perennial shrub or small tree in tropical and subtropical region in outer Himalayas regions with an altitude below 1,000 m and are reported to have wide range of pharmacological activities.* Mallotus philippinensis* species are known to contain different natural compounds, mainly phenols, diterpenoids, steroids, flavonoids, cardenolides, triterpenoids, coumarins, isocoumarins, and many more especially phenols; that is, bergenin, mallotophilippinens, rottlerin, and isorottlerin have been isolated, identified, and reported interesting biological activities such as antimicrobial, antioxidant, antiviral, cytotoxicity, antioxidant, anti-inflammatory, immunoregulatory activity protein inhibition against cancer cell. We have selected all the pharmacological aspects and toxicological and all its biological related studies. The present review reveals that* Mallotus philippinensis* is a valuable source of medicinally important natural molecules and provides convincing support for its future use in modern medicine. However, the existing knowledge is very limited about* Mallotus philippinensis* and its different parts like steam, leaf, and fruit. Further, more detailed safety data pertaining to the acute and subacute toxicity and cardio- and immunotoxicity also needs to be generated for crude extracts or its pure isolated compounds. This review underlines the interest to continue the study of this genus of the Euphorbiaceae.

## 1. Introduction

*Mallotus* (family: Euphorbiaceae) is a large genus of trees and shrubs distributed chiefly in the tropical and subtropical regions of the Old World with around 20 species in India [1].* Mallotus philippinensis* Muell. (commonly called Kamala, Kampillaka, and Kapila, and locally known as Shendri) is a very common perennial shrub or small tree found in outer Himalayas ascending to 1500 meters. Mature fruits have glandular hairs collected as reddish brown powder which is collected in cloth by shaking and rubbing the fruits by hand. The collected material is fine, granular powder, dull red, or madder red-colored and floats on water. This plant is traditionally used for antifilarial [2], antibacterial, anti-inflammatory, and immune-regulatory activity [3] and also used as purgative, anthelmintic, vulnerary, detergent, maturant, carminative, alexiteric and is useful in treatment of bronchitis, abdominal diseases, spleen enlargement, antimicrobial, antiparasitic, and so forth (Figure 18).

Some other medicinal plants reported similar anticestodal activity shown in Table 1. Some medicinal plants exported from India are* Aconitum* species (root),* Acorus calamus* (rhizome),* Adhatoda vasica* (whole plant),* Berberis aristata* (root),* Cassia angustifolia *(leaf and pod),* Colchicum luteum* (rhizome and seed),* Hedychium spicatum* (rhizome), and* Heradeum candicans* (rhizome) [4].

### 1.1. Scientific Classification

Consider the following:

 kingdom: Plantae,
 subkingdom: Tracheobionta,
 superdivision: Spermatophyta,
 division: Magnoliophyta,
 class: Magnoliopsida,
 subclass: Rosidae,
 order: Euphorbiales,
 family: Euphorbiaceae,
 genus:* Mallotus*,
 species:* Mallotus philippinensis.*

### 1.2. Botanic Description

Trees are small to medium-sized monoecious in nature, up to 25 m tall and with a bole up to 50 cm in diameter, but usually much less in number (Figure 19(a)). Slash turning deep red. Branchlets are reddish-brown glandular.

Leaves are alternate and simple, more or less leathery, ovate to lanceolate, cuneate to rounded with two glands at base. Leaves are mostly acute or acuminate at apex, conspicuously 3-nerved, hairy and reddish glandular beneath, petiole size 1–4 cm long, puberulous and reddish-brown in color (Figure 19(b)). Male flowers in terminal and axillary position, 2–10 cm long, solitary or fascicled paniculates spikes, each flowers are with numerous stamens, small; female flowers have spikes or slender racemes, each flower with a stellate hairy, 3 celled ovary with 3 papillose stigmas. Fruit is a depressed-globose; 3-lobed capsule; 5, 7 mm, and 10 mm; stellate; puberulous; with abundant orange or reddish glandular granules; 3-seeded (Figure 19(d)). Seeds are subglobose and black in color and 4 mm across (Figure 19(e)) [5].

### 1.3. Biology

In this genus,* Mallotus philippinensis* (*M. philippinensis)* flowers mature from March to April and fruits mature in July-August.* M. philippinensis* has extra floral nectaries attracting ants.

### 1.4. Ecology

*M. philippinensis* has a widespread natural distribution, from the western Himalayas, through India, Sri Lanka, to southern China, and throughout Malesia to Australia. Sometimes it is gregarious but more usually mixed with other species, both in forests and open scrubland. Kamala tree is common in evergreen forest, especially in secondary forest, and sometimes even dominant in the undergrowth. Kamala tree withstands considerable shade; it is frost-hardy and resistant to drought.

### 1.5. Biophysical Limits

Kamala tree is mostly grown at an altitude of 0–1600 m at a mean annual temperature of 16–28°C with mean annual rainfall of 800–2000 mm. Plants will grow mostly in a wide range of soil types, including infertile soils, limestone, acid, and rocky land.

## 2. Vernacular Names and Traditional Uses

### 2.1. Vernacular Names

The vernacular names are as follows: English: Kamala tree; Hindi:* Kamala, Sindur, Rohini*, and* Kambhal*; Bengali:* Kamala, Kamalagundi*; Guajarati:* Kapilo*; Kannad:* Kampillaka, Kunkumadamara*; Malayalam:* Sinduri, Manjana, Kuramatakku, Kampipala, and Ponnagam*; Marathi:* Shindur, Shendri, and Kapila*; Punjabi:* Kumila, Kamal, Kambal, and Kamela*; Tamil:* Kapli, Kungumam, Kurangumanjanatti, Kamala, Manjanai, Kunkumam, and Kamala*; Telugu:* Kunkuma, Chendra-sinduri, Kapila, Vassuntagunda, Sundari, Vasanta, and Kumkumamu*; Arabic:* Kinbil*; Assam:* Gangai, Puddum, and Lochan*; Oriya:* Bosonto-gundi, Kumala, Sundragundi, and Kamalagundi*; Pers:* Kanbela*; Santhal:* Rora*.

### 2.2. Traditional Uses

According to Ayurveda, leaves are bitter, cooling, and appetizer. All parts of plant like glands and hairs from the capsules or fruits are used as heating, purgative, anthelmintic, vulnerary, detergent, maturant, carminative, and alexiteric and are useful in treatment of bronchitis, abdominal diseases, and spleen enlargement, and if taken with milk or curd (yoghurt), it can be quite useful for expelling tapeworms [6]. Kamala or Kampillakah is also used as an oral contraceptive. The powder and a few other parts of Kamala are also used in external applications to promote the healing of ulcers and wounds. They are used to treat parasitic affections of the skin like scabies, ringworm, and herpes.

### 2.3. Common Adulterants

Glandular hair powder of* M. philippinensis* is commonly adulterated with Annato dye (*Bixa orellana *Linn.), ferric oxide, brick dust, and ferruginous sand.* Casearia tomentosa* (stem bark powder),* Carthamus tinctorius* (flower powder),* Ficus benghalensis* (fruit powder), and* Flemingia macrophylla* (hairs of fruits) are also reported to be used as adulterant or substitute of Kampillaka [7].

## 3. Chemical Constituents

Major phytochemicals present in this genus contain different natural compounds, mainly phenols, diterpenoids, steroids, flavonoids, cardenolides, triterpenoids, coumarin, isocoumarins, and many more to discover. Present knowledge about this endangered species of medicinal plant is still limited with respect to its phytochemistry and biological activity. However, some researchers have contributed towards isolation of some novel constituents and their activity. One of the major chemical constituent, that is, rottlerin of* M. philippinensis,* is listed below with its chemical structure and its major biological activities along with other phytochemicals (Figure 18).

### 3.1. Cardenolides

*M. philippinensis* seeds contain cardenolides. The seeds of* M. philippinensis* were found to contain after fermentation four cardenolides (Table 2), of which two were newly reported: corotoxigenin L-rhamnoside and corogl-aucigenin L-rhamnoside [8].

### 3.2. Triterpenoids

**Figure 1:**
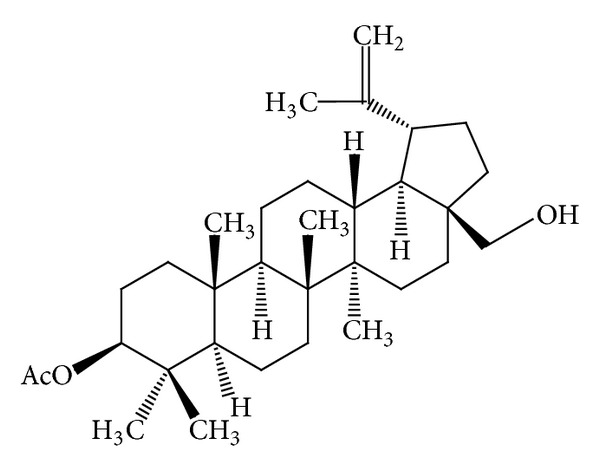
Betulin-3-acetate.

**Figure 2:**
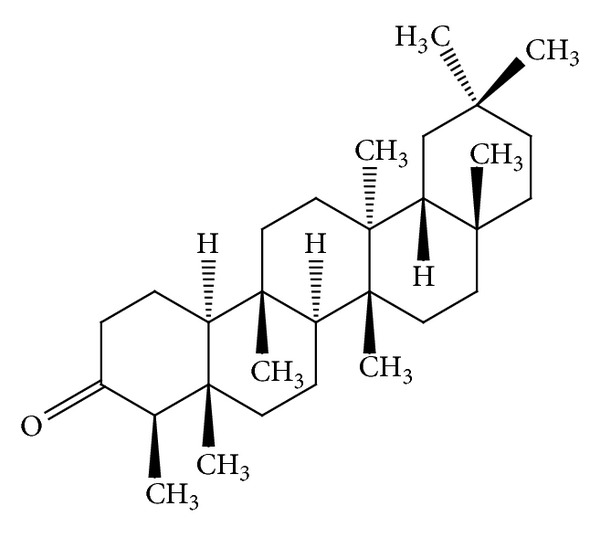
Friedelin.

**Figure 3:**
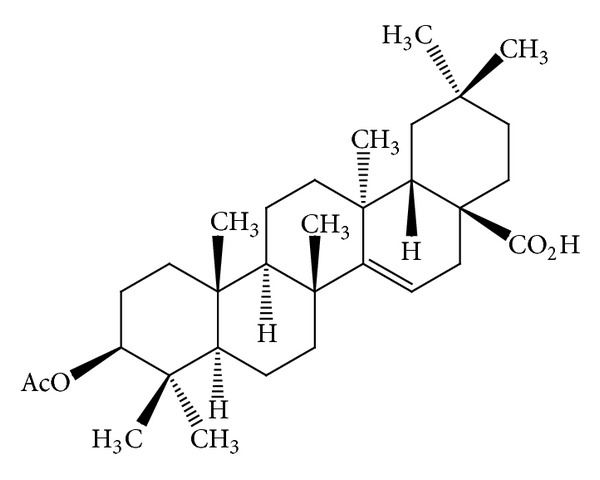
Acetylaleuritolic acid or aleuritic acid acetate.

**Figure 4:**
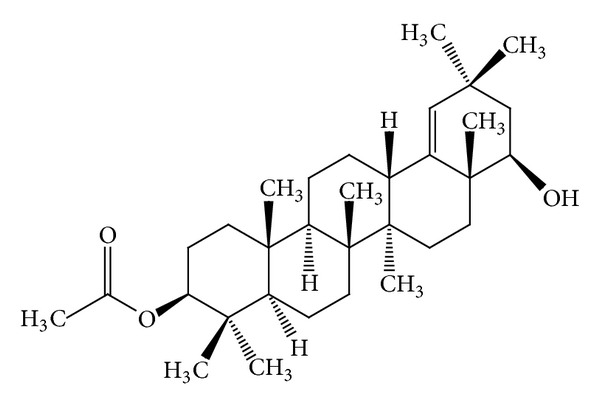
3b-Acetoxy-22b-hydroxyolean-18-ene.

**Figure 5:**
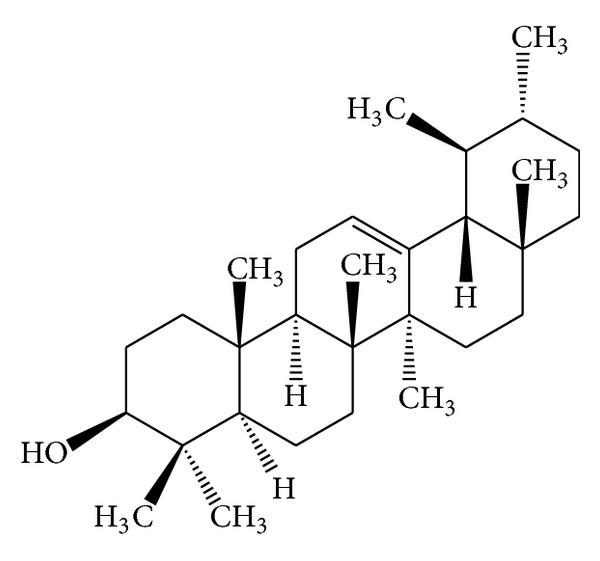
*α*-Amyrin.

Some* Mallotus* species were found to have a characteristic feature of pentacyclic triterpenoids with a 6/6/6/6/5 ring system. The petroleum ether extract of the heartwood of* M. philippinensis* yielded triterpenoids: betulin-3-acetate (Figure 1) as a major compound, lupeol acetate (Table 3), and lupeol (Table 3) [9]. Friedelane-type triterpenoids are very common in* Mallotus* species. Friedelin (Figure 2) was obtained from the stem bark of* M. philippinensis *[10]. Most of the genera of family Euphorbiaceae, such as Drypetes [11] or Celaenodendron [12] also contain friedelin in rich amount. Friedelin is common and is also found in plants from other orders. Other known pentacyclic terpenoids, that is, acetylaleuritolic acid (Figure 3) found to be reported in the petroleum and ether extracts from bark of* M. philippinensis *[9]. The first olean-18-ene triterpene oxidized at C-22 (Figure 4) was isolated from the stem bark of* M. Philippinensis *[10]. Apart from above triterpeniods, ursane-type triterpenoid: a-amyrin (Figure 5) reported from the petroleum ether and ethereal extracts of* M. philippinensis* bark [9].

### 3.3. Steroids

**Figure 6:**
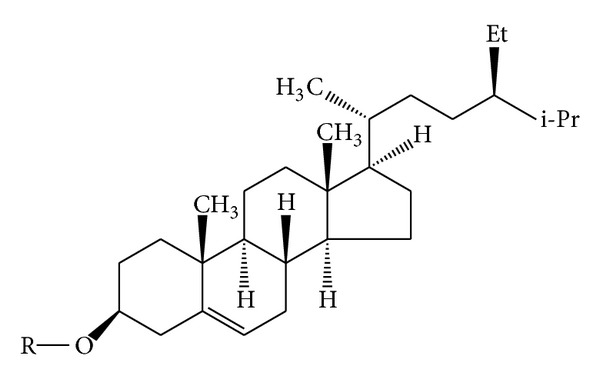
*β*-Sitosterol.

Common steroid, b-sitosterol (Figure 6), was isolated from petroleum ether extracts of the heartwood and bark of* M. philippinensis *[9]. Daucosterol was obtained from ether extract from bark of* M. philippinensis *[9].

### 3.4. Phenolic Compounds

**Figure 7:**
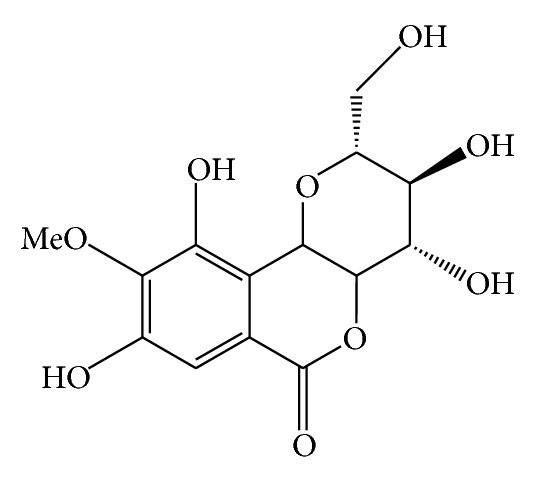
Bergenin.

Isocoumarins, Bergenin (Figure 7), an isocoumarin, was isolated in 1972 from the heartwood of* M. philippinensis*. This compound was also obtained from the bark and the leaves of* M. philippinensis *[9].

**Figure 8:**
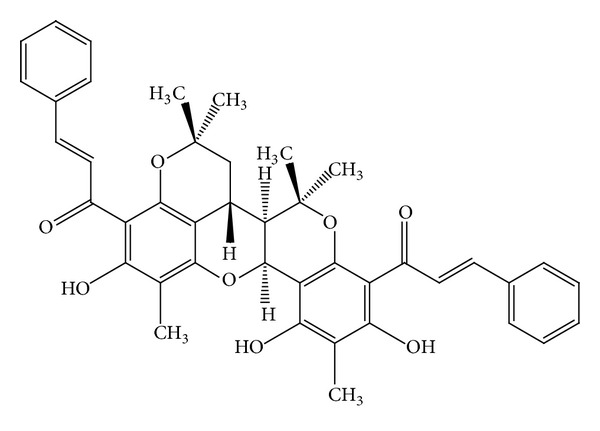
Kamalachalcone A.

**Figure 9:**
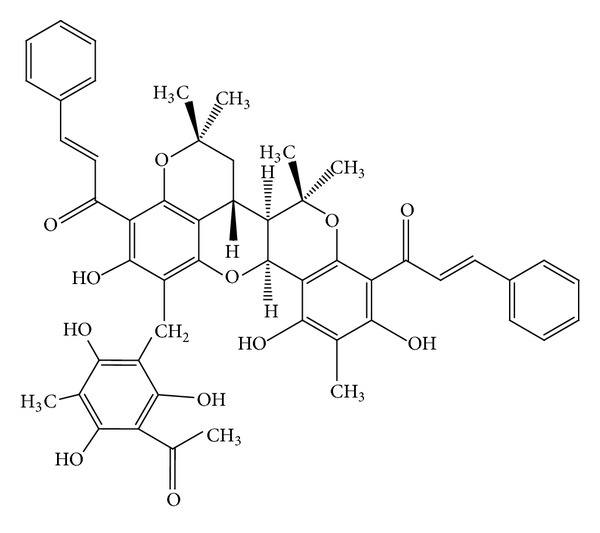
Kamalachalcone B.

**Figure 10:**
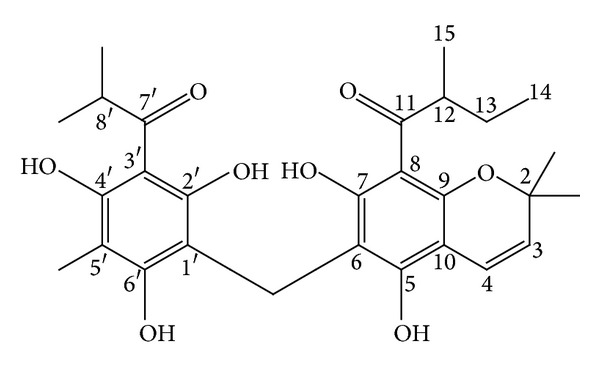
Mallotophilippen A or 1-[5,7-dihydroxy-2,2-dimethyl-6-(2,4,6-trihydroxy-3-isobutyryl-5-methyl-benzyl)-2H-chromen-8-yl]-2-methyl-butan-1-one.

**Figure 11:**
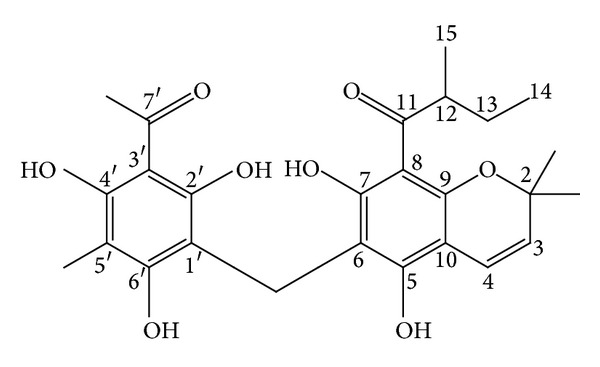
Mallotophilippen B or 1-[6-(3-acetyl-2,4,6-trihydroxy-5-methyl-benzyl)-5,7-dihydroxy-2,2-dimethyl-2H-chromen-8-yl]-2-methyl-butan-1-one.

**Figure 12:**
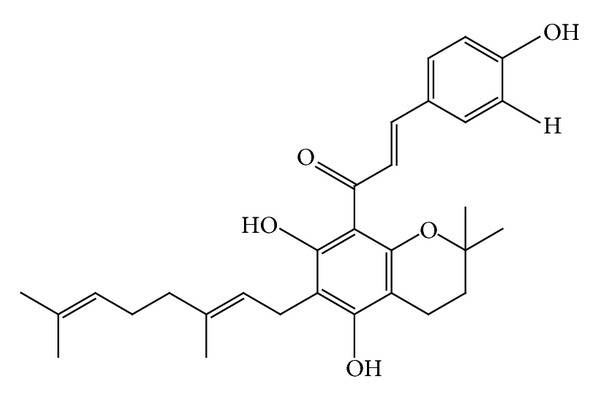
Mallotophilippen C or (1-[6-(3,7-dimethyl-octa-2,6-dienyl)-5,7-dihydroxy-2,2-dimoethyl-2H-chromen-8-yl]-3-(4-hydroxy-phenyl)-propenone), R = H.

**Figure 13:**
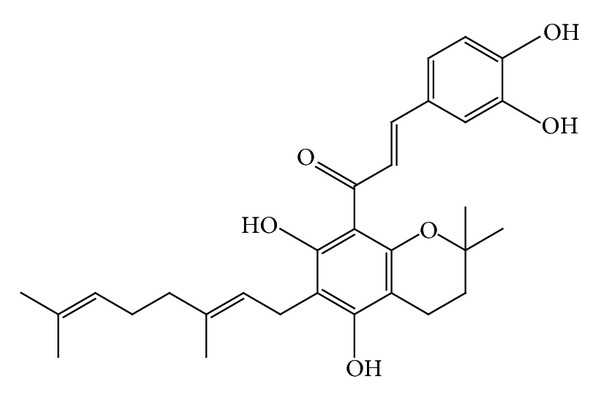
Mallotophilippen D or 3-(3,4-dihydroxy-phenyl)-1-[6-(3,7-dimethyl-octa-2,6-dienyl)-5,7-dihydroxy-2,2-dimethyl-2H-chromen-8-yl]-propenone, R = OH.

**Figure 14:**
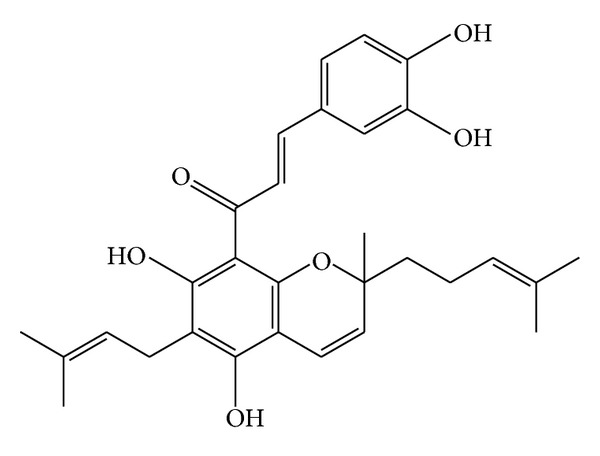
Mallotophilippen E or 1-[5,7-dihydroxy-2-methyl-6-(3-methyl-but-2-enyl)-2-(4-methyl-pent-3-enyl)-2H-chromen-8-yl]-3-(3,4-dihydroxy-phenyl)-propenone.

Two new chalcone derivatives (flavonoids), kamalachalcones A and B (Figures 8-9) with a unique ring system caused by dimerization between a dimethylchromene ring and a phenoxyl group, were isolated from kamala (*M. philippinensis*) [13]. Three other novel chalcone derivatives, mallotophilippens C, D, and E (Figures 10, 11, 12, 13 and 14), were isolated from the fruits of* M. philippinensis *[14], lignans, chalcones, and dimeric chalcone derivatives [15].

**Figure 15:**
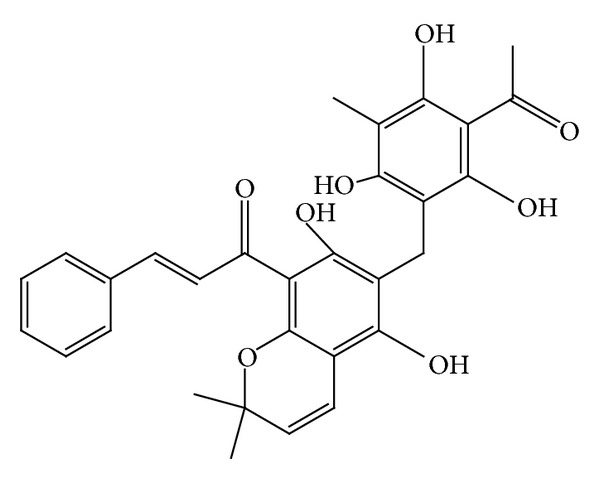
Rottlerin.

**Figure 16:**
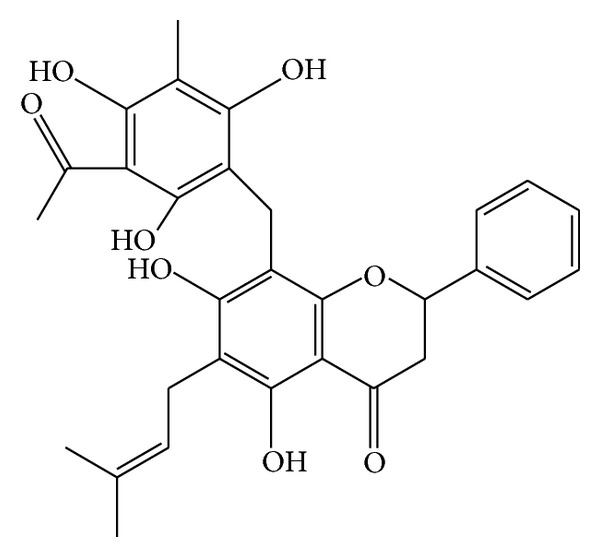
Isoallorottlerin.

**Figure 17:**
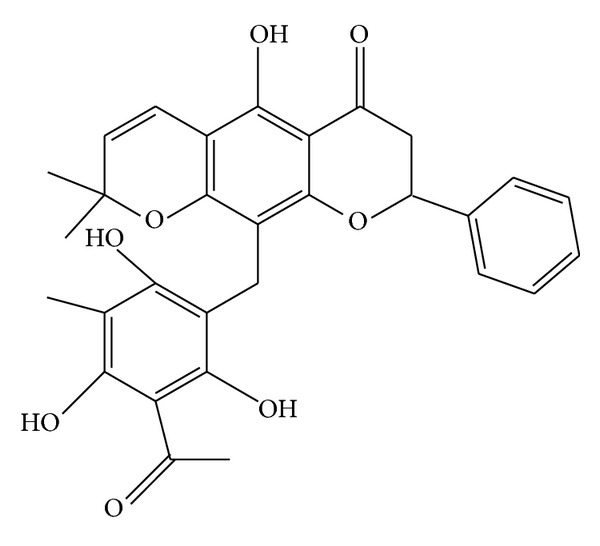
Isorottlerin.

**Figure 18:**
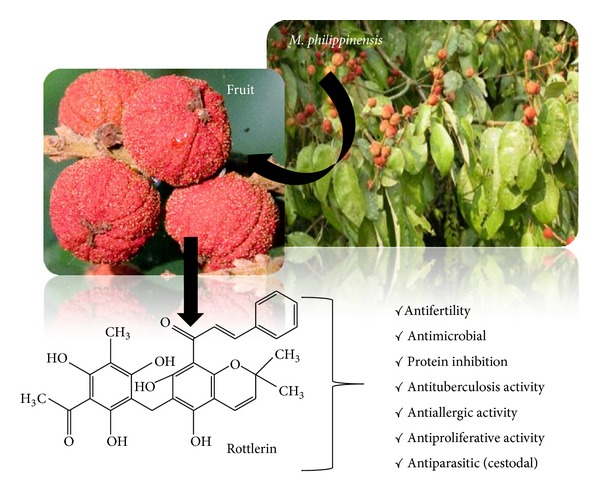
Rottlerin and its major biological activities.

**Figure 19:**
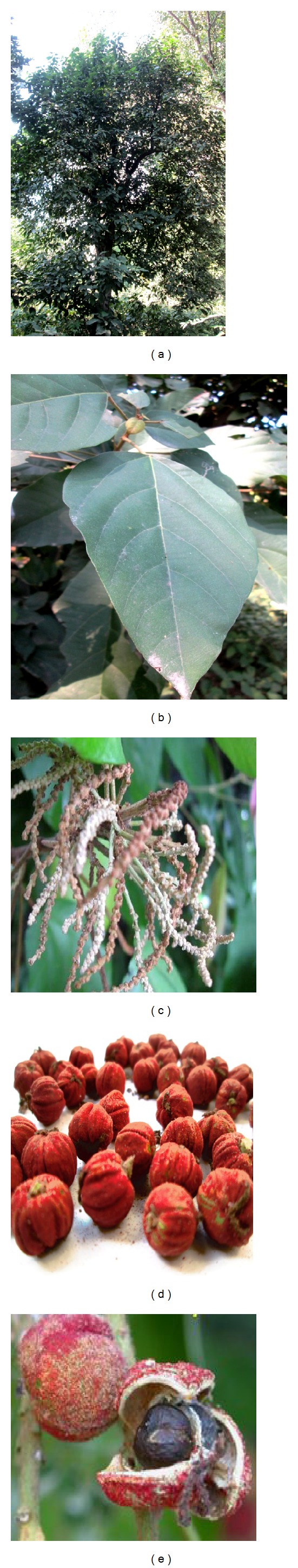
*Mallotus philippinensis.* (a) Mature plant; (b) leaf; (c) initial inflorescence of seed setting; (d) mature fruits twig; (e) mature fruit with seed.

Four phloroglucinol derivatives (kamalins), rottlerin, and isorallorottlerin (Figures 15 and 16) were isolated from* M. philippinensis* [16]. Isorottlerin (Figure 17) was also mentioned in* M. philippinensis *[17].

Fruit and bark of plant contain condensed tannins responsible for antioxidant activity. Methanolic bark extract of* M. philippinensis* subjected to characterization through column chromatography on a Sephadex LH-20 column using ethanol and acetone-water as the mobile phases, tannins and phenols were quantified. Bark extract contains 541 mg/g of total phenolics and infractions ranging from 54 mg/g (fraction I) to 927 mg/g (fraction VI) and condensed tannins were detected in fractions II–VI [18, 19]. In 1989, leaves of* M. philippinensis* were reported for tannins and other related compounds by Saijo et al. [20].

### 3.5. Other Compounds

Unsaturated fatty acids, that is, triplyunsatured hydroxy acid kamlolenic acid, different fatty acids, and glyceride [21] have been reported from Kamala (*M. philippinensis*) seed oil.

Resinous coloured material contains active parts of rottlerin and isorottlerin. It also contains homorottlerine, red role 50%, yellow role 5%, manure 2%, volatile oils, tannin, gum, citric acid, and oxalic acid.

## 4. Pharmacological Activities

### 4.1. Antifilarial Activity

The effect of aqueous and alcoholic leave extracts of* M. philippinensis* (Lam.) Muell. was studied on the spontaneous movements of the whole worm and nerve-muscle (n.m.) preparation of* Setaria cervi* and on the survival of microfilariae* in vitro*. Both the extracts result in inhibition of spontaneous motility of whole worm and the n.m. preparation of* S. cervi* characterized by initial stimulation followed by depression in amplitude. The tone and rate of contractions remained visibly unaffected. Aqueous extract at higher concentration showed immediate reduction in tone. The concentration required to inhibit the movements of n.m. preparation was 1/5th for aqueous and 1/11th for alcoholic extract compared to that for the whole worm, suggesting a cuticular permeability barrier. The stimulatory response of acetylcholine was blocked by aqueous extract on whole worm movements. On the microfilariae the LC_50_ and LC_90_ were 18 and 20 ng/mL for aqueous and 12 and 15 ng/mL for alcoholic extracts, respectively [2]. Further study will be required to evaluate the same activity with its phytochemicals.

### 4.2. Antifertility Activity

Seeds extract of* M. philippinensis* exhibits adverse effects on different reproductive parameters of female rats. According to the study, extract reduces serum FSH and LH levels, probably by affecting hypothalamic/pituitary axis in experimental animals. This reduced level may affect follicular development, quality of ovulated eggs, corpus luteum formation, estrus cycle, and maintenance of pregnancy in rats [22]. This antifertility effect of plant extract is supposed to be caused by rottlerin (Figure 15), a phloroglucinol derivative. Acetyl rottlerin may be active, but isorottlerin (Figure 17) is either inactive or slightly active [23]. Effect of pure rottlerin can be further studied so as to clarify the potential of phloroglucinol derivatives.

### 4.3. Antibacterial and Antifungal Activity

A series of 61 Indian medicinal plants belonging to 33 different families used in various infectious disorders were screened for their antimicrobial properties. Screening was carried out at 1000 and 500 *μ*g/mL concentrations by agar dilution method against* Bacillus cereus* var* mycoides*,* Bacillus pumilus, Bacillus subtilis, Bordetella bronchiseptica, Micrococcus Luteus, Staphylococcus aureus, Staphylococcus epidermidis, Escherichia coli, Klebsiella pneumonia, Candida albicans, and Saccharomyces cerevisiae*. Twenty-eight plant extracts showed activity against at least one of the test organisms used in the screening. On the basis of the results obtained, study concludes that the crude extracts of* M. philippinensis* exhibited significant antimicrobial activity [3] and properties that support folkloric use in the treatment of some diseases as broad-spectrum antimicrobial agents. Steam bark of plant and its chloroform fractions and the methanolic extract significantly inhibit the pathogenic bacteria with significant zones of inhibition comparable to the standard drug used. However, the hexanic extract did not show any significant activity [24]. Glandular hair of fruits of* Mallotus* exhibits significant antibacterial activity against human pathogenic bacteria with MIC ranging 15–20 mg/mL. This extract does not show any inhibition against different species of* candida*. This shows that fruit extract possesses antibacterial activity without any antifungal potential. The results of the study may justify the use of the plant against bacterial pathogens. This probably explains the use of these plants by the indigenous people against a number of infections [25].

However, ethanolic extract shows potent anti-*Helicobacter pylori* activity at the concentration of 15.6–31.2 mg/L against eight* H. pylori* strains. Further purification of extract revealed that rottlerin exhibits potent bactericidal effect with minimal bactericidal concentration (MBC) of 3.12–6.25 mg/L against different resistant strains of clarithromycin and metronidazole including Japanese and Pakistani strains [17].

### 4.4. Anti-Inflammatory and Immunoregulatory Activity

Chalcones derivatives from the fruits of* M. philippinensis and* mallotophilippens C, D, and E (Figures 12, 13, and 14) inhibit nitric oxide (NO) production and inducible NO synthase (iNOS) gene expression by a murine macrophage-like cell line (RAW 264.7), which was activated by lipopolysaccharide (LPS) and recombinant mouse interferon-gamma (IFN-gamma). Further investigations suggest the downregulation of cyclooxygenase-2 gene, interleukin-6 gene, and interleukin-1b gene expression. The above results show that these chalcones have good anti-inflammatory and immunoregulatory effects [26].

### 4.5. Antioxidant Activity and Antiradical Activity

Different fractions of bark and fruit of* Mallotus* were studied for its total antioxidant activity (TAA) and antiradical activity against DPPH on a Sephadex LH-20 column using ethanol and acetone-water as mobile phase. Among different extracts, bark fraction showed the strongest antiradical activity (TAA value—5.27 mmol Trolox equiv./g) and reducing power. Another extract, that is, phenolic fraction, shows TAA ranging from 0.58 mmol Trolox/g (fraction I) to 6.82 mmol Trolox/g (fraction IV); this is the strongest fraction showing antiradical activity against DPPH and reducing power. TAA of other extracts ranged from 0.05 to 1.79 mmol Trolox equiv./g [18, 19].

### 4.6. Protein Inhibition Implicated in Cancer Processes

Protein kinase is inhibited with some specificity for PKC by rottlerin, a compound isolated from* Mallotus*. Inhibition of PKC appears due to a strong competition between rottlerin and ATP. CaM-kinase III is suppressed by rottlerin as effectively as PKC *δ*, among different protein kinases tested. Novel inhibition property and improved selectivity for a distinct PKC isoenzyme of rottlerin are suggestive from its chemical structure [27, 28]. Rottlerin is also very potent in blocking other kinases including Akt/PKB and p38 MAPK [29–31]. It also inhibits human T cell responses [32], reduces MUC5AC expression in human epithelial cells [33], abrogates reactive oxygen species production in hepatic stellate cells [34], and prevents histamine-induced H1-receptor gene expression in HeLa cells [35]. However, still very limited information is available of rottlerin towards cancer disease and its mechanism of action.

### 4.7. Hepatoprotective Activity

Methanolic extract of* M. philippinensis* leaves decreases the CCl_4_-induced elevation in biochemical parameters (SGOT, SGPT, SALP, direct bilirubin, total bilirubin, and MDA) on pretreatment at doses 100–200 mg/kg and also reversed the functional and antioxidant parameters. This study suggests that leave extract was effective in functional improvement of hepatocytes. Histopathological studies also suggest the hepatoprotective activity of plant [36].

### 4.8. *In Vitro* Cytotoxicity against Human Cancel Cell

Glandular hair extract of* Mallotus* fruit powder was assayed against 14 human cancer cell lines among different fractions; 95% ethanolic extract showed the highest cytotoxic effect as compared to 50% ethanolic and aqueous portion. Further, the chromatographic analysis of the said fraction afforded a polyphenolic molecule rottlerin in* Mallotus* plant [37].

### 4.9. Anticestodal Activity/Veterinary Applications

*M. philippinensis* fruit was found to be very effective against gastrointestinal cestodes in Beetal goats and other ruminants. Comparative anticestodal efficacies of single oral dose treatments with the powdered fruit of* M. philippinensis* (125, 250, and 375 mg/kg), its water or methanol extracts (equivalent to 375 mg/kg), and the total glycosides (25, 50, and 100 mg/kg) were determined in naturally cestode-infected Beetal goats [38]. An ethnobotanical survey has been conducted for anthelmintics in ruminants, so as to document the plants used to treat and control helminthes.* Mallotus* has been frequently used to treat helminthosis in ruminants [39].* M. philippinensis* fruit extract of 800 mg/kg twice daily for 3 days was observed to have curative efficacy against mature adult worms of* Hymenolepis diminuta*. The total follow-up period of 90 days did not show any further excretion of eggs in the faeces of treated rats. Praziquantel at the dosage of 5 mg/kg also produced a similar effect [40].* In vitro* scolicidal activity of* M. philippinensis* (Lam.) Muell Arg. fruit glandular hair extract against hydatid cyst* Echinococcus granulosus *protoscoleces at concentrations 10 and 20 mg/mL shows the mortality rate 97% to 99%, respectively, for 60 min treatment, while up to 93% mortality was observed with 20 mg/mL for only 10 min treatment. This proves that the extract has significant scolicidal activity with almost no associated side effects [41].* In vivo* animal model experiment will be further required to prove its effect against Hydatid cyst.

### 4.10. Purgative Activity and Anthelmintic Activity

A significant purgative effect after an oral dose (120 mg/kg) in rats was assessed from resins isolated from plant. Its effect was evaluated from the weight of faeces as well as from surface area of blotting paper soaked by liquid faeces. The anthelmintic effect on tape worm was evaluated in albino rates, from the resin of the plant showed lethal effect of 35.69% and 78.21% respectively in small intestine in concentrations 60 and 120 mg/kg respectively [42, 43].

### 4.11. Antituberculosis Activity

Organic extract of plant after bioassay-directed fractionation yields five compounds, the most active of which against* Mycobacterium tuberculosis* was a new compound, 8-cinnamoyl-5,7-dihydroxy-2,2-dimethyl-6-geranylchromene for which the name mallotophilippen F is suggested. The second compound 8-cinnamoyl-2,2-dimethyl-7-hydroxy-5-methoxychromene was isolated from a natural source for the first time, while the remaining three compounds, rottlerin, isoallorottlerin, or isorottlerin and the so-called “red compound,” 8-cinnamoyl-5,7-dihydroxy-2,2,6-trimethylchromene, had been already isolated from this plant. Isolated compounds were identified by 2D-NMR and C-13 NMR [44]. Ethanolic extract of plant was assayed for antimycobacterial activity against* M. smegmatis* by disc diffusion assay. Further antituberculosis potential of leaves extract was identified by radiometric BACTEC assay; result revealed that ethanolic extract of* M. philippensis* showed antituberculosis activity against virulent and avirulent strains of* M. tuberculosis* H37Rv and* M. tuberculosis* H37Ra with minimum inhibitory concentrations of 0.25 and 0.125 mg mL^−1^, respectively. The inhibition in growth index values of* M. tuberculosis* was observed in the presence of ethyl acetate fraction at a minimum concentration of 0.05 mg mL^−1^. It suggests that ethanolic and ethyl acetate fraction of plant possesses significant antimycobacterial activity [45]. Steam bark of* M. philippinensis* has also been reported for its antitumor promoting effect, which was due to the presence of 3*α*-Hydroxy-D:A-friedooleanan-2-one [46].

### 4.12. Antiallergic Activity

*M. philippinensis* fruit contains two new phloroglucinol derivatives, mallotophilippens A and B (Figures 10 and 11) which were identified, using chemical and spectral data, as 1-[5,7-dihydroxy-2,2-di-methyl-6-(2,4,6-trihydroxy-3-isobutyryl-5-methyl-benzyl)-2H-chromen-8-yl]-2-methyl-butan-1-one and 1-[6-(3-Acetyl-2,4,6-trihydroxy-5-methyl-benzyl)-5,7-dihydroxy-2,2-dimethyl-2H-chromen-8-yl]-2-methyl-butan-1-one, respectively. These compounds inhibited the production of nitric oxide (NO) and inducible NO synthase (iNOS) gene expression by a murine macrophage-like cell line (RAW 264.7), which was activated by lipopolysaccharide (LPS) and recombinant mouse interferon-g (IFN-g). Further, phloroglucinol derivatives inhibit histamine release from rat peritoneal mast cells induced by compound 48/80. This study suggests its anti-inflammatory activity [47]. Rottlerin has been tested in animal models of IgE-dependent anaphylaxis and the antiallergic mechanisms of action in mast cells. Antiallergic action of rottlerin has been tested in passive cutaneous anaphylaxis and passive systemic anaphylaxis mouse models and in anaphylactic contraction of bronchial rings isolated from sensitized guinea pigs. This experiments proves antiallergic effect of rottlerin by blocking IgE-induced mast cell degranulation. This report suggests the use of rottlerin in mast cell-mediated allergic disorders including urticaria and allergic asthma [48].

### 4.13. Anti-Leukaemic Activity

Root extract of* M. philippinensis* was tested on human promyelocytic leukemia HL-60 cell proliferation, cell cycle regulators, and apoptosis in order to investigate its antileukemic effect. Hexane fraction showed promising toxicity against p53-deficient HL-60 cells (IC_50_ 1.5 mg dry roots equivalent/mL medium) after 72 h and, interestingly, inhibition of cell proliferation was preceded by the upregulation of the protooncogenes Cdc25A and cyclin D1 within 24 hours suggesting its antileukemic effect in HL-60 cells. After isolation and identification by GC-MS, polyphenols were the main compounds of the hexane extract that inhibited proliferation and induced apoptosis [49].

### 4.14. Antiproliferative Activity

Antiproliferative effect was evaluated against Thp-1 cell lines from the isolated compounds of* M. philippinensis* fruit extract, in which 4′-hydroxyrottlerin showed 54% growth inhibition of Thp-1 cell line [50]. Other isolated compounds were also tested against different fungi and were found to be very effective IC_50_ values.

### 4.15. Anti-HIV Activity

Four phloroglucinol derivatives, named mallotophenone (5-methylene-bis-2,6-dihydroxy-3-methyl-4-methoxyacetophenone), mallotochromene (8-acetyl-5,7-dihydroxy-6-(3-acetyl-2,4-dihydroxy-5-methyl-6-methoxybenzyl)-2,2-dimethylchromene), mallotojaponin (3-(3,3(dimethylallyl) S-(3(acetyl-2,4-dihydroxy-5-methyl-6-methoxybenzyl)-phloracetophenone), and mallotolerin (3-(3-methyl-2-hydroxybut-3-enyl)-5-(3-acetyl-2,4-dihydroxy-5-methyl-6-methoxybenzyl)-phloracetophenone), were tested for their ability to inhibit the activity of human immunodeficiency virus- (HIV-) reverse transcriptase. The mode of inhibition of mallotojaponin was found to be competitive with respect to the template primer, (rA)n (dT)12–18, and noncompetitive with respect to the triphosphate substrate, dTTP. The K_*i*_ value of mallotojaponin for HIV-reverse transcriptase was determined to be 6.1 *μ*M [51].

### 4.16. Antitumor Activity

Four known friedelane-type triterpenoids, friedelin, 3-hydroxy-D:A-friedoolean-3-en-2-one, 2*β*-hydroxy-D:A-friedooleanan-3-one, and 3*α*-hydroxy-D:A-friedooleanan-2-one, and two known lupane-type triterpenoids, lupeol and betulin, were isolated from the stem bark of* M. philippinensis* and were tested for their inhibitory effects on Epstein-Barr virus early antigen (EBV-EA) activation induced by 12-O-tetradecanoylphorbol 13-acetate (TPA). The inhibitory effect of compounds 2 (IC_50_ = 292 mol ratio/32 pmol/TPA) and 4 (IC_50_ = 288) was stronger than those of the other compounds tested and the positive control, curcumin (IC_50_ = 343). Compound 3*α*-hydroxy-D:A-friedooleanan-2-one strongly inhibited mouse skin tumor promotion in an* in vivo* two-stage carcinogenesis model [46].

### 4.17. Wound Healing and Mesenchymal Stem Cell (MSC) Proliferation

Bark extract of* Mallotus philippinensis* has been tested* in vitro* for wound healing activity by examining the proliferation and migration of MSCs. KUM6 cells proliferation and migration have been enhanced at 0.16–4 *μ*g/mL and unregulated the activity of MSCs by secreting various cytokines to wounded site from bone marrow to systemic circulation and finally remodel wounded tissues [52].

### 4.18. Toxicities

Seeds of* M. philippinensis* ethereal extract have adverse effect on various parameters of female rats. Even the extract reduces serum levels of gonadotropins in treated animals at high dose of 100 mg/kg body weight. Reduced weights of ovary and uterus, follicular development, and increased atretic follicular in the ovary are due to subnormal levels of steroid hormones. Thus, pregnancy is very difficult in female rats treated with kamala seed extract [22].

### 4.19. Colouring Agent-Dye

Glandular hairs of fruit are mostly used as an orange dye for silk [53, 54].

## 5. Pharmacognostic Evaluation of* Mallotus philippinensis*

Morphological study shows that fruit depresses globose and is three-lobed capsule, 5–7 mm × 8–10–12 mm, stellate-puberulose, and with abundant orange or reddish glandular granules. Seeds are subglobose and black in color. Organoleptic property of the red fruit shows that it is tasteless and odourless. Microscopic description showed the presence of epicarp, which contained a compactly packed layer of mucilaginous cells, and mesocarp composed of columnar cells which are closely arranged. Its polygonal cells are compactly arranged in 2-3 layers. Presence of lignified vascular arrangement has been observed in the transverse section [55].

## 6. Conclusion and Future Perspective

Medicinal plants have been clinically used and its interest has been dramatically increased over the past decades throughout the world and its formulations are increasingly cited in media. Daily consumption of the natural products and their formulations by an extensive number of patients lead to serious concern for scientist to study its efficacy and safety. Because of extensive use and its benefits, natural products in many countries are regulated both as medicinal products and as food supplements, often labeled as natural food supplements.

Traditional use and its growing demand for* Mallotus philippinensis* and its other species lead to compile this review and commented on the current knowledge provided by clinical and preclinical research on the effect of this plant.

*Mallotus philippinensis* has been widely used as traditional medicine in several parts of countries including India. Every part of this plant possesses its specific medicinal properties and is used mainly in ayurveda to fight against intestinal worms in domestic and grazing animals when administered with jaggery. However, only a few reports are attributed to this plant and its different parts and there is a large scope for investigation. Hence, it is required to explore more of its potential within the field of medicinal and pharmaceutical sciences for novel and fruitful application of this plant in form of natural formulation. Along with this medicinal importance, this plant is used against human pathogens including* H. pylori*, anti-inflammatory activity, antioxidant, antiradical, protein inhibition, hepatoprotective, antiallergic, anti-HIV activity, and many more. Phytochemical investigation revealed that a large number phenol derivatives and several miscellaneous compounds from different classes have been isolated from this species. The phenols, diterpenoids, steroids, flavonoids, cardenolides, triterpenoids, coumarins, and isocoumarins are mostly distributed in all parts of the plant. The other major isolated pure compounds from this species mostly belong to phenolic group exhibiting most of the biological activity. Various types of extracts from different parts and single compounds derived from this species have been found to possess biological activities, including antioxidant, antimicrobial, anti-inflammatory, cytotoxicity, and immune modulatory. Fruit and bark of plant contain condensed tannins responsible for antioxidant activity. Some novel chalcone derivatives, mallotophilippens C, D, and E, were isolated from the fruits of* M. philippinensis*. Mallotoxin or rottlerin has great anticancerous potential. Among the ever-anticancer agents, rottlerin appears to have great potentiality for being used in chemotherapy. Rottlerin will become a potential molecule for research in future to treat cancerous cell as it will affect cell machineries involved in apoptosis, survival, and autophage. This suggests the view that this species has potential to be a beneficial chemotherapeutic remedy.

Although the data and other reports provided that this medicinal plant is of great biological use in different pharmacological activities including anticancer, further research is needed in different areas regarding the toxicity and efficacy of pure phytochemicals isolated from different parts of this plant. More data will be needed from preclinical and clinical studies on humans to clarify its potency and safety, as lack of knowledge with respect to its adverse effects and methodological accuracy in the literature limits towards its standardized formulation. Furthermore, the mechanism of action of the phytochemicals and extract of* Mallotus philippinensis* is unclear; more exhaustive studies will be performed to explore its mechanism and structure activity relationship among various constituents.

In conclusion, this review confirms the great potential of* Mallotus philippinensis*. As very limited information is still known for this species, it leads us to continue the study on different species of* Mallotus* plant and its interesting pharmacological properties. Further natural product chemistry of isolated moiety and its structural analysis of compounds responsible for these activities will be an interesting field of research.

## Figures and Tables

**Table 1:** Some medicinal plants reported for the antihelmintic activity.

S. no.	Sources	Part used	Family	Reference
1	*Strobilanthes discolor *	Leaves	Acanthaceae	[56]
2	*Trifolium repens *	Aerial shoot	Fabaceae	[57]
3	*Houttuynia cordata* Thumb	Leaves	Piperaceae	[58]
4	*Lasia spinosa* Linn	Leaves, stalk, stem	Araceae	[59]
5	*Centella asiatica* Linn	Leaves	Apiaceae	[60]
6	*Clerodendrum colebrookianum* Walp	Leaves	Verbenaceae	[61]
7	*Gynura angulosa* DC	Leaves	Asteraceae	[61]
8	*Aloe vera* Linn	Leaves	Liliaceae	[61]
9	*Psidium guajava* Linn	Leaves	Myrtaceae	[62]
10	*Curcuma longa* Linn	Rhizomes	Zingiberaceae	[63]
11	*Ocimum sanctum* Linn	Oil/eugenol	Lamiaceae	[64]
12	*Albizia anthelmintica *	Steam bark	Mimosaceae	[65]
13	*Berlinia grandiflora *	Steam bark	Leguminosae	[66]
14	*Nicotiana tabacum* L.	leaves	Solanaceae	[67]
15	*Calotropis procera* (Ait.) Ait.	Flowers	Asclepiadaceae	[68]

**Table 2:** Cardenolide and its derivatives.

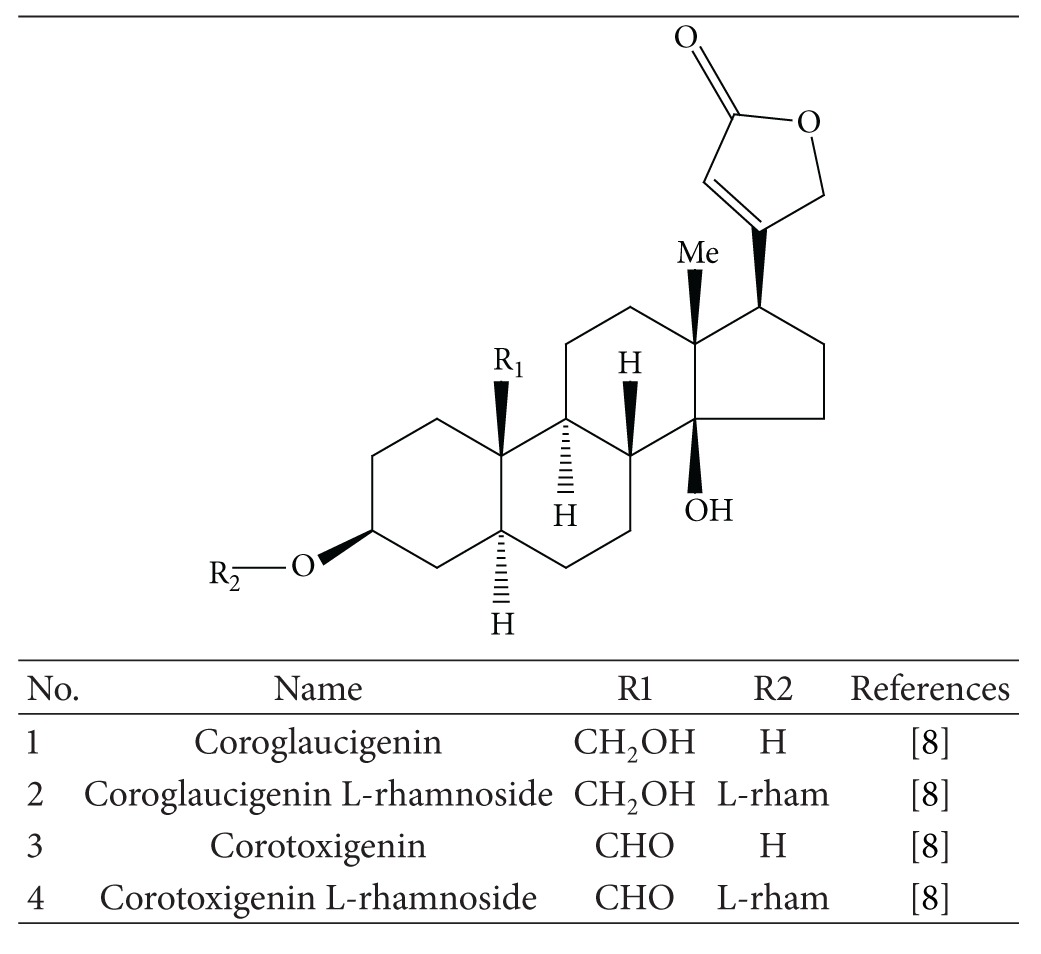

**Table 3:** Lupeol and its derivatives.